# Supplementary material for: Exact inversion of partially coherent dynamical electron scattering for picometric structure retrieval
Source: Nat Commun. 2024 Jan 2;15:101. doi: 10.1038/s41467-023-44268-x (PMC10762228; doi:10.1038/s41467-023-44268-x)
Supplement: Supplementary file 1 — Supplementary Information [file 41467_2023_44268_MOESM1_ESM.pdf]

Supplementary Information to:

Exact inversion of partially coherent dynamical electron scattering for picometric structure retrieval

Content

Supplementary Note 1	The inverse problem in electron diffraction	1
Supplementary Note 2	Relative tilt between illumination and specimen	2
Supplementary Note 3	Partial spatial and temporal coherence	2
Supplementary Note 4	Implementation of Frozen Phonon Layers	3
Supplementary Note 5	Model fit for partial coherence	4
Supplementary Figure 1	Pixelwise reconstruction with varying cutoff angle (simulation study)	4
Supplementary Figure 2	Initial and reconstructed phase gratings for the theoretical study	5
Supplementary Figure 3	Comparison of experimental and reconstructed diffraction patterns	5
Supplementary Figure 4	Loss versus iteration epoch for $PbZr_{0.2}Ti_{0.8}O_3$ experiment	6
Supplementary Figure 5	Loss versus specimen thickness in $PbZr_{0.2}Ti_{0.8}O_3$ experiment	6
Supplementary Figure 6	$PbZr_{0.2}Ti_{0.8}O_3$ structure from Debye-Waller approximation	7
Supplementary Table 1	Starting values for the displacements in $PbZr_{0.2}Ti_{0.8}O_3$.	7
Supplementary References		7

Supplementary Notes

Supplementary Note 1 The inverse problem in electron diffraction

The (coherent) reconstruction problem in ptychography can be described by determining the specimen $\mathcal{S}$ and the incoming wave ϕ from phaseless recordings

$$I(\mathbf{s}) = |\mathcal{F}[M(\phi(\cdot - \mathbf{s}), \mathcal{S})]|^2 .$$

Here, $\mathbf{s} \in \mathbb{R}^2$ is the scan position, $\mathcal{F}$ the Fourier transform, taking the exit wave to the detector plane, and M the interaction model. It describes the interaction between the specimen $\mathcal{S}$ and the incoming wave ϕ. Classically, direct methods usually assume that the scan positions lay on a regular grid. Furthermore, they work with a highly simplified interaction model, like single-sideband [1] (using a weak phase object approximation) and Wigner Distribution Deconvolution [2] (which uses a multiplicative transmission function).

More complex models or scan grids have to be approached differently. Here, iterative methods are the prime candidates. While the family of PIE algorithms [3, 4] (ptychographic iterative engine, an iterative projection-based algorithm) was used extensively, over the last few years a new prime challenger appeared in the form of gradient-based methods [5, 6]. They proved capable of optimizing more advanced models, as outlined in the

following. The first important improvement is using a multislice interaction model. It splits the specimen into multiple slices and calculates their projected potentials $V_1, \ldots, V_S$ to obtain

$$M(\phi, V_1, \ldots, V_S) = e^{i\sigma V_S} \cdot (F \otimes (e^{i\sigma V_{S-1}} \ldots (F \otimes (e^{i\sigma V_1} \cdot \phi)))). \tag{S.1}$$

Here, F is the Fresnel propagator, which propagates the wave by the distance between the current and the next slice. The propagator multiplies the wave in the Fourier domain with

$$P(k, \Delta z, \theta) = e^{-i\pi\lambda k^2 \Delta z + 2\pi i \Delta z (k_x \tan\theta_x + k_y \tan\theta_y)}. \tag{S.2}$$

Here, θ_x, θ_y is the specimen tilt and Δz the slice distance. This model describes the coherent forward scattering problem in exact manner, however, established inverse techniques reconstruct the phase of the exponentials without physical constraints so far.

The second improvement is mixed-state ptychography, which addresses partial coherence. As our approach differs significantly from previously used formulations, we discuss it in more detail in the next section. Most important for the work presented here are the methods relying on an atomic model. Furthermore, we chose a different approach to address the limited initial knowledge about the probing wave ϕ. While often the discretized wave is optimized pixelwise, an approach called blind ptychography, we use a semi-blind approach, where we parametrize ϕ by a few aberration parameters. In our results we optimized for defocus C_1, 2- and 3-astigmatism A_1 and A_2, coma B_2 and spherical aberration C_S.

Supplementary Note 2 Relative tilt between illumination and specimen

All free parameters are optimized via the automatic differentiation routine of Pytorch. For some, like aberration parameters, this is straight forward, as they are used only at a single place, the probe generation. However, other parameters occur in multiple places, most importantly the tilt (another example are atomic positions in models periodized in z direction). For the tilt, we use $\Theta = (\tan\theta_x, \tan\theta_y)$ as free parameteres, and the function that is differentiated can be thought of as

$$\Theta \mapsto \underbrace{(\Theta, \ldots, \Theta)}_{S-1 \text{ times}} \mapsto (P(k, \Delta z, \Theta), \ldots, P(k, \Delta z, \Theta)).$$

These $S - 1$ values are then plugged into the Fresnel propagators F in (S.1). Standard multivariate calculus shows that the gradient induced in Θ is the sum of the gradients induced in the single slices. Note that these gradients scale automatically with the slice thickness, therefore for a given total thickness of the specimen, the learning rate is invariant with respect to different slicing schemes. This approach differes in that regard from the parametrization in [7, 8], where only the gradient with respect to the middle slice is considered.

Supplementary Note 3 Partial spatial and temporal coherence

Partial spatial coherence can be modelled by a density function ρ such that

$$I(\mathbf{s}) = \int_{\mathbb{R}^2} \rho(\mathbf{p} - \mathbf{s}) \left|\mathcal{F}[M(\phi(\cdot - \mathbf{p}), \mathcal{S})]\right|^2 \mathrm{d}\mathbf{p}\,. \tag{S.3}$$

Instead of an integral, in simulations one typically chooses a suitable quadrature rule and approximates

$$I(\mathbf{s}) \approx \sum_{k=1}^{K} \beta_k \left|\mathcal{F}[M(\phi(\cdot - \mathbf{s} + \eta_k), \mathcal{S})]\right|^2 .$$

However, we usually do not know the density ρ exactly and can thus also not pick a suitable quadrature formula for the integral (S.3). That is why we optimize for the weights β_k and offsets η_k as well during the inversion procedure. The energy spread of the incoming electrons, as well as electron-optical instabilities lead to partial temporal coherence. It can be modelled by letting the defocus of the probe vary slightly in different simulations, and by summing the resulting intensities. The same reasoning as above applies and we end up with an additional offset for the defocus of ϕ. In that respect, these parametrizations allow to estimate the incoherence parameters.

To demonstrate that our approach to partial coherence during the inversion of the problem in physically parametrized manner is reliable, we applied it to the data published by Chen et al. [9]. The results are shown below. Negative potential values have been set to zero after a 50 episodes, then the reconstruction continued iterating for 50 more episodes. Also, a regularization of the form $\|\mathcal{F}T\|_1$, where T is complex-valued phase grating (representing $e^{i\sigma V}$ in an ideal case) was used. This should be contrasted to the phase unwrapping and the so-called missing wedge regularization used by Chen et al. [9] Further adaptations, e.g. also employing the missing wedge regularization, could lead to even better results.

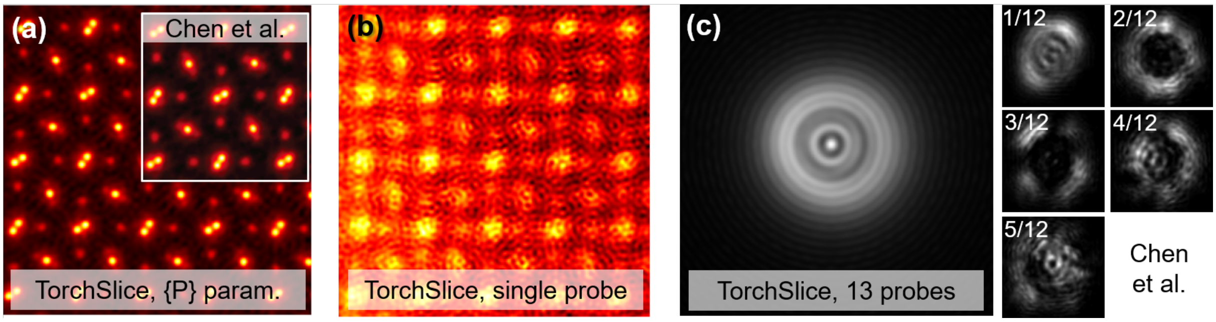

Reconstruction from the dataset of Chen et al. a) Our reconstruction, using 13 offsets for the probes. Reconstruction from Chen et al. is shown for comparison. b) Using just a single probe will not lead to a satisfying reconstruction, demonstrating the necessity of incorporating incoherence. c) shows our probe (calculated from the recovered aberration coefficients, exactly as the algorithm does it) and the first five modes recovered by Chen et al. We use the same probe, up to a defocus and position offset) for each of the 13 incoherently summed exit waves.

Supplementary Note 4 Implementation of Frozen Phonon Layers

The potential as stated in formula (1) in the article is implemented as follows. The Fourier transforms of v_{Z_n}, denoted by A_{Z_n}, is precalculated for each type occurring in the specimen. Enumerating the types with $l = 1, \ldots, L$ one can calculate the potential with a sum over the L types as follows:

$$V_{\text{FP}}^{(j)} = \mathcal{F}\left[\sum_{l=1}^{L} A_l \odot \left[\exp(-2\pi i(p_x \cdot (x^l)^T)) \cdot \operatorname{diag}(w^l) \cdot \exp(-2\pi i(y^l \cdot p_y^T))\right]\right].$$

Here, $p_x = (kh_1)_k \in \mathbb{R}^K$ and $p_y = (kh_2)_k \in \mathbb{R}^K$ are the Fourier frequencies, with equispaced sampling of step size h_1 and h_2 (in practice, we choose both equal in all examples). x^l, y^l are vectors containing all x resp. y coordinates of the atoms of type l, modified by the random offset. $\operatorname{diag} w^l$ is a diagonal matrix, containing the weights of the atoms of type l. $\odot$ denotes the pointwise product. Note that $p_x \cdot (x^l)^T$ is an outer product, then the matrix product between the two exponential terms is formed. The gradients, with respect to x^l, y^l and w^l, are computed by the automatic differentiation routine in Pytorch.

Supplementary Note 5 Model fit for partial coherence

The proposed model of Verbeeck et al. [10] of the density for spatial partial coherence as introduced in (S.3) is given by

$$\rho(r) = \frac{2\pi ac}{(c^2 + 4\pi^2 r^2)^{2/3}} + (1-a)\frac{\pi}{d^2} e^{-\pi^2 r^2/d^2}.$$

Fitting to our data yielded $a = 0.171,\ c = 2.31,\ d = 6.11$, parameters comparable to the findings in Ref. [10]. An exact match is not expected, as they depend on the microscope used and further parameters such as the spot number and gun lens setting. It should be noted that the weights of the quadrature formula are not directly comparable to samples of the density. Also, the number of quadrature points is too low for any reasonable fit. This result can only be understood qualitatively in that sense that the distribution is more heavy-tailed than a Gaussian and could be better covered by a model including a Cauchy distribution than using a Gaussian only.

Supplementary Figures

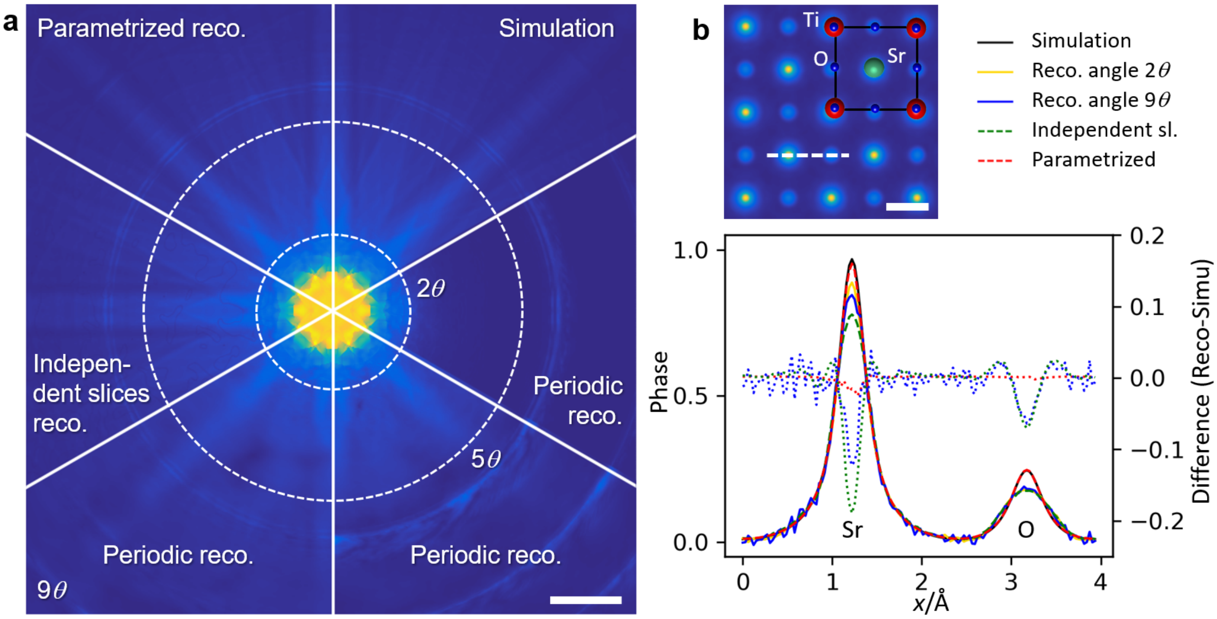

Supplementary Figure 1: Impact of thermal diffuse scattering on pixelwise reconstructions by means of PACBED. **a.** Reconstructions were performed for a simulated 4D-data set of 20 nm thick $SrTiO_3$ including 50 FP configurations for the forward simulation. Periodic reconstructions employed a single phase grating reused in each slice, which was reconstructed pixelwise for reciprocal space cutoffs of 2, 5 and 9 times the semi-convergence angle θ. Although TDS cannot be included physically correct in this approach, the diffuse intensity in the diffraction patterns can formally be reconstructed. Additionally, a pixelwise reconstruction assuming no specimen periodicity in beam direction (independent slices) has been performed, yielding the TDS details nearly perfectly without evident differences to a parametrized, FP-based inversion shown top left. Scale bar, 50 mrad. **b.** However, respective line profiles (bottom) in reconstructed real-space phases across the Sr-O unit (dashed white line, top) differ significantly. Here, the pixelwise reconstruction schemes, including the independent slice model, underestimate the dynamic range of the phases with respect to the ground truth (black solid line) that was the input for the simulation. Moreover, TDS causes noisy phases in the reconstruction for periodic slice models. A parametrized reconstruction in which atomic potentials have been initialised by fitting atomic weights w_n and positions a previous pixel-wise reconstruction achieved accurate final phases (dashed red line) in congruence with the ground truth. Scale bar, 2 Å.

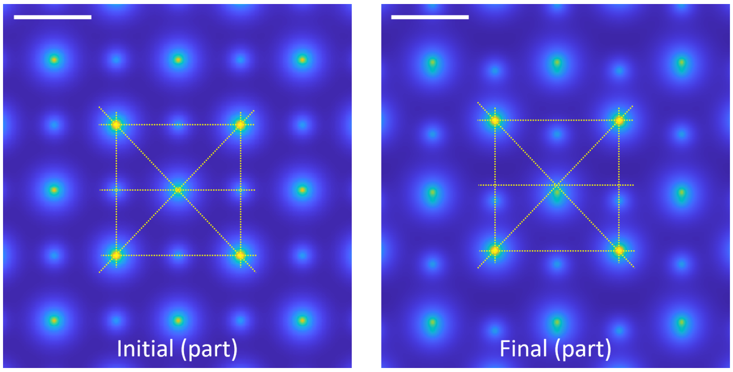

Supplementary Figure 2: Comparison of initial and reconstructed phase gratings for $PbZr_{0.2}Ti_{0.8}O_3$ (part). The main article shows the result of the reconstructed potential in Fig. 2. Here, the phase grating according to the atom positions in the initial model is shown on the left, to be compared with the result after reconstruction on the right in a region of 3×3 unit cells. Atoms have been put at symmetry positions in the initial model, not imposing any polarisation as indicated by the yellow lines. The corners of the inset mark Pb atoms. For better simultaneous visibility of light and heavy atom columns, the square root of the phase is shown. Scale bar, 5 Å.

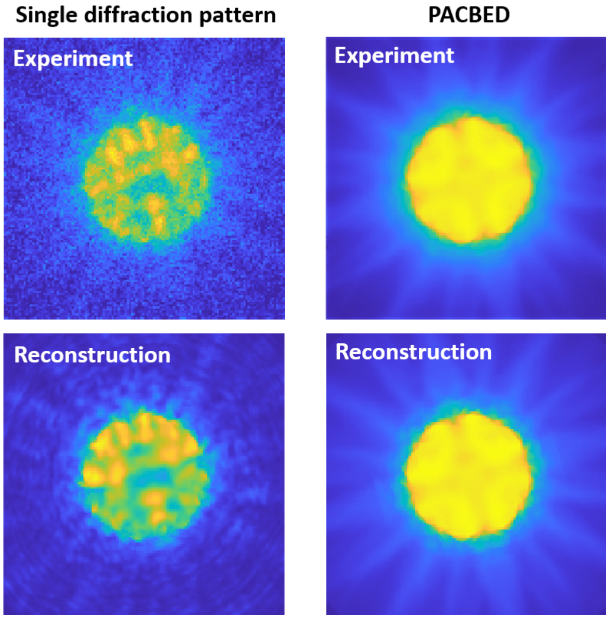

Supplementary Figure 3: Comparison of experimental and reconstructed diffraction patterns. For the final reconstruction of the experimental 4D-STEM data of $PbZr_{0.2}Ti_{0.8}O_3$, the figure shows recorded raw diffraction patterns in the top row and the ones that have been simulated from the inverted model according to the final epoch. The left column shows an exemplary single diffraction pattern, the position-averaged diffraction pattern is depicted in the right column.

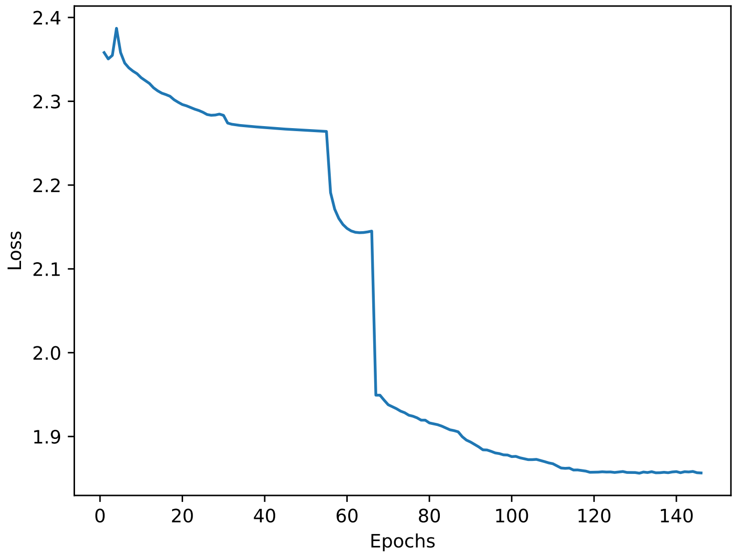

Supplementary Figure 4: Loss of the experimental dataset. The first drop (epoch 55) is due to an increase of resolution of the potentials, the second (epoch 68) due to switching to the frozen phonon model.

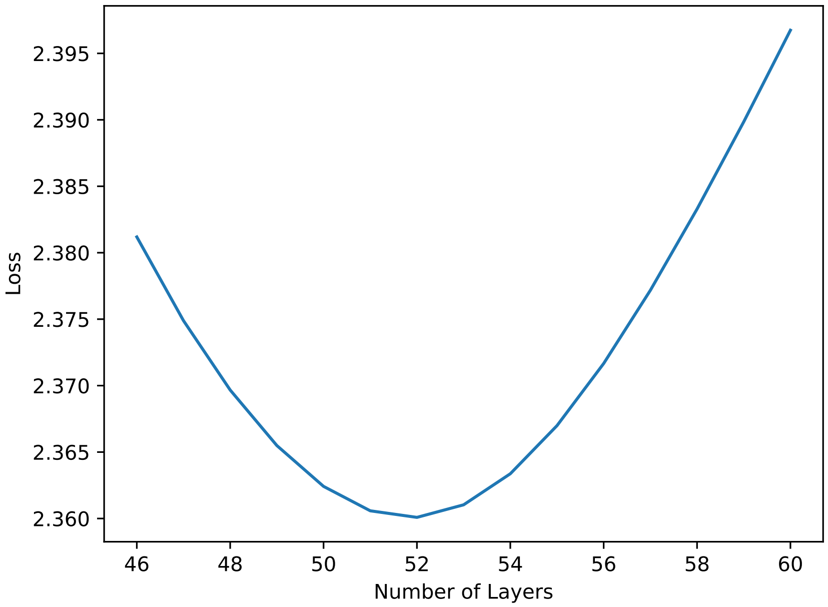

Supplementary Figure 5: Loss for different numbers of layers using the initial atomic model. A clear minimum is visible at 52 layers, each of thickness equal to one unit cell, which was used in all subsequent models.

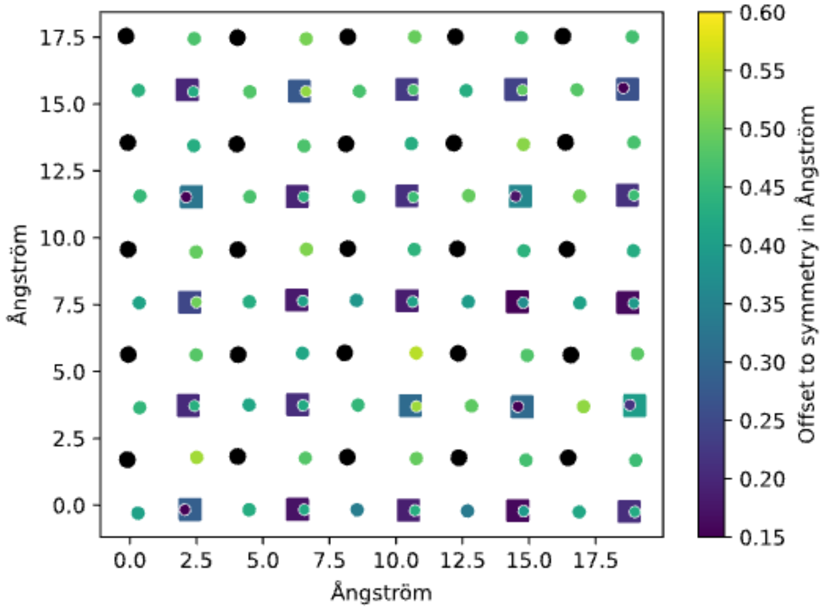

Supplementary Figure 6: Experimental polarisation measurement in ferroelectric $PbZr_{0.2}Ti_{0.8}O_3$ using a single state model with Debye-Waller approximation.

Supplementary Table 1: Starting values for the displacements in $PbZr_{0.2}Ti_{0.8}O_3$. Offset of the atomic columns along both crystal axis from the symmetry position, averaged over the 25 unit cells in the reconstructed region. One can see that the starting values already indicate the polarization direction, however they are quite far from the literature values from Ref. [11]. The standard deviation is given in brackets.

**Site**	**c direction** Exp. start [pm]	Lit. [pm]	**a direction** Exp. start [pm]	Lit. [pm]
Ti/Zr	15 (6)	17/26	−7 (5)	0
O_1	20 (6)	43	−10 (4)	
O_2	30 (6)	48	2 (7)	
O_3	15 (6)	48	−7 (5)	

Supplementary References

1. Rodenburg, J. M., McCallum, B. C. & Nellist, P. D. Experimental tests on double-resolution coherent imaging via STEM. *Ultramicroscopy* **48**, 304–314 (1993).

2. Rodenburg, J. M. & Bates, R. H. T. The Theory of Super-Resolution Electron Microscopy Via Wigner-Distribution Deconvolution. *Phil. Trans. A: Phys. Sci. Engin.* **339**, 521–553 (1992).

3. Maiden, A. M. & Rodenburg, J. M. An improved ptychographical phase retrieval algorithm for diffractive imaging. *Ultramicroscopy* **109**, 1256–1262 (2009).

4. Maiden, A. M., Humphry, M. J. & Rodenburg, J. M. Ptychographic transmission microscopy in three dimensions using a multi-slice approach. *Journal of The Optical Society of America A-optics Image Science and Vision* **29**, 1606–1614 (2012).

5. Schloz, M. *et al.* Overcoming information reduced data and experimentally uncertain parameters in ptychography with regularized optimization. *Optics Express* **28**, 28306–28323 (2020).

6. Van den Broek, W. & Koch, C. T. Method for retrieval of the three-dimensional object potential by inversion of dynamical electron scattering. *Physical Review Letters* **109**, 245502 (2012).

7. Sha, H., Cui, J. & Yu, R. Deep sub-angstrom resolution imaging by electron ptychography with misorientation correction. *Science Advances* **8**, eabn2275 (2022).

8. Sha, H. *et al.* Sub-nanometer-scale mapping of crystal orientation and depth-dependent structure of dislocation cores in $SrTiO_3$. *Nature Communications* **14**, 162 (2023).

9. Chen, Z. *et al.* Electron ptychography achieves atomic-resolution limits set by lattice vibrations. *Science* **372**, 826–831 (2021).

10. Verbeeck, J., Béché, A. & Van den Broek, W. A holographic method to measure the source size broadening in STEM. *Ultramicroscopy* **120**, 35–40 (2012).

11. Frantti, J. *et al.* Neutron Diffraction Studies of $Pb(Zr_xTi_{1-x})O_3$ Ceramics. *Japanese Journal of Applied Physics* **39**, 5697–5703 (2000).
