# Supplementary material for: Exact inversion of partially coherent dynamical electron scattering for picometric structure retrieval
Source: Nat Commun. 2024 Jan 2;15:101. doi: 10.1038/s41467-023-44268-x (PMC10762228; doi:10.1038/s41467-023-44268-x)
Supplement: Supplementary file 3 — Description of Additional Supplementary Files [file 41467_2023_44268_MOESM3_ESM.pdf]

Description of Additional Supplementary Files:

Supplementary Software 1: Source code for multislice forward simulation, and parametrized as well as pixel-based inverse multislice reconstruction as presented in the main article.
